# Prevalence of Disability Among Older Adults in Prison

**DOI:** 10.1001/jamanetworkopen.2024.52334

**Published:** 2024-12-27

**Authors:** Katherine E. M. Miller, Karen Shen, Yang Yang, Brie A. Williams, Jennifer L. Wolff

**Affiliations:** 1 Department of Health Policy and Management, Bloomberg School of Public Health, The Johns Hopkins University, Baltimore, Maryland; 2 Roger and Flo Lipitz Center to Advance Policy in Aging and Disability, The Johns Hopkins University, Baltimore, Maryland; 3 Hopkins Economics of Alzheimer’s Disease & Services Center, The Johns Hopkins University, Baltimore, Maryland; 4 Priscilla Chan and Mark Zuckerberg San Francisco General Hospital and Trauma Center, San Francisco, California; 5 UCSF Division of Health Equity and Society, University of California, San Francisco

## Abstract

**Importance:**

The number of older adults in long-term correctional facilities (prisons) has increased rapidly in recent years. The cognitive and functional status of this population is not well understood due to limitations in the availability of longitudinal data.

**Objective:**

To comparatively examine the prevalence and disability status of the population of adults 55 years and older in prisons and adults living in community settings for a 14-year period (2008-2022).

**Design, Setting, and Participants:**

This repeated cross-sectional study used nationally representative data from the American Community Survey to identify cohorts of community-dwelling adults 55 years or older and adults 55 years or older in prisons. Data were accrued from surveys from 2008 to 2022 and analyzed from August 25, 2023, to October 18, 2024.

**Exposure:**

Residence in federal or state prison.

**Main Outcomes and Measures:**

Self-reported cognitive, ambulatory, independent living, or self-care difficulties.

**Results:**

The population of older adults in prisons increased between 2008 and 2022 from 102 700 to 171 700, but the pace of growth slowed during the COVID-19 pandemic. The analysis included 32 623 adults 55 years or older in prison (mean [SD] age, 62.6 [8.3] years; 28 927 [89.64%] male) and 13 877 665 living in the community (mean [SD] age, 67.4 [9.5] years; 7 497 784 [54.02%] female). Disability prevalences in all categories, including cognitive, ambulatory, independent living, and self-care difficulties, were significantly higher compared with their community-dwelling peers. The difference was particularly stark for cognitive difficulty, which was 15.2% (95% CI, 14.8%-15.7%) for older adults in prisons vs 7.1% (95% CI, 7.1%-7.2%) in the community, after adjusting for demographic characteristics. These prevalences have remained stable over the sample period, including the COVID-19 pandemic.

**Conclusions and Relevance:**

Results of this cross-sectional study suggest that adults in prison are more likely to report disability than those in community settings, with the largest differences in cognitive difficulties. These differences persisted during the COVID-19 pandemic. The notable increase among older adults in prisons coupled with the high prevalences of disability has important implications for identifying care models and environmental policies to address the chronic and end-of-life care needs of this population, particularly within the context of constrained resources of health care in prisons.

## Key Points

**Question:**

What is the prevalence of cognitive and functional limitations among the aging population in prison?

**Findings:**

In this cross-sectional study of 32 623 individuals 55 years or older in prisons, disability prevalence was significantly higher compared with 13 877 665 older individuals in the community after adjusting for demographic characteristics. The difference was particularly stark for cognitive difficulty among older adults in prisons, at 15.2% vs 7.1%.

**Meaning:**

These findings have important implications for identifying care models and environmental policies to address the chronic and end-of-life care needs of this population, particularly within the context of constrained resources of health care in prisons.

## Teaser

This cross-sectional study compares prevalence of disability among adults 55 years or older in prison with that of community-dwelling peers and examines changes in the prevalence before and during the COVID-19 pandemic among the prison population.

## Introduction

The prison population is aging. The number of people in prison who are 55 years and older grew by 280% from 1999 to 2016, compared with just 3% for the population younger than 55 years.^1^ In 2020, nearly one-third of people with life sentences were older than 55 years of age.^2^ The aging of the population in prison (long-term correctional facilities, eg, for individuals with sentences of >1 year, vs short-term facilities such as jails) has been mostly attributed to the large population first imprisoned in the 1970s and 1980s as the rate of incarceration of the US population more than quadrupled between 1970 and 2009.^3^ Causes of increased incarceration during this era included more punitive sentencing (eg, mandatory minimum sentences and 3-strikes laws) and intensified policing and enforcement of drug laws.^3^

Providing care to an aging population in prison poses significant challenges to the criminal legal system. It is twice as expensive to imprison older (vs younger) individuals,^4,5,6^ and the built environment and available resources are often misaligned with needs resulting from age-related disability such as cognitive impairment, functional limitations, or hearing loss.^5,7,8^ The vulnerability of this population and the challenges of providing care in prisons were evident during the COVID-19 pandemic, during which rates of deaths in prisons surpassed deaths in nursing homes and rates of compassionate release from federal prisons increased, particularly for older adults.^9,10^

Data on the demographic characteristics and health status of people who are in prison are sparse.^6^ The primary source of this information comes from the Survey of Prison Inmates, conducted by the Bureau of Justice Statistics. The survey is conducted at irregular intervals (most recently, 2016), and questions about health status are heavily slanted toward substance use and mental health.^11^ Consequently, studies of disability to date have primarily relied on qualitative methods or primary data collection at individual facilities with limited generalizability and small samples.^6,12^ Collectively, the absence of longitudinal data on the health status of older adults in prison is a barrier to identifying and addressing the evolving care needs of this vulnerable population.

The aim of this study was to use nationally representative longitudinal data and methods to strengthen our understanding of the prevalence of disability among older adults in prisons over time, both prior to and during the COVID-19 pandemic, with foundational information for evidence-based planning and policy.^3,5^ We developed an algorithm that draws on publicly available data to identify a population of older adults likely in prison. Then, we described the prevalence of disability among older adults in prison, in contrast with community-dwelling peers. Finally, we examined changes in prevalence of disability of the likely population in prison prior to and during the COVID-19 pandemic. Understanding these changes is critical to interventions and policies addressing this population.

## Methods

### Data Sources and Sample

We use data from the American Community Survey (ACS) from 2008 through 2019 and 2021 through 2022. The ACS is a nationally representative survey conducted by the US Census Bureau annually.^13^ We excluded the year 2020 because the data collection of the ACS was disrupted due to the pandemic, and survey weights for 2020 are considered experimental. We identified cohorts of community-dwelling adults 55 years and older and adults 55 years and older in prison. While the threshold of 65 years is most often used to distinguish older adults, 55 years is more often used to characterize older adults in prison due to accelerated aging in this context.^5,12^ Because we used publicly available data, this study did not constitute human participants research as defined at 45 CFR §46.102 and thus was exempt from institutional review board oversight and informed consent. We followed the Strengthening the Reporting of Observational Studies in Epidemiology (STROBE) reporting guideline.

To identify the population likely in prison, we first limited the sample to individuals living in institutional settings, defined as nursing homes, jails, or prisons in the ACS. Because publicly available data do not distinguish type of institutional residence (ie, nursing home vs prison), we leveraged variation in regulations that exclude individuals in prison from receiving certain federal benefits to delineate types of residential setting. Specifically, we identified individuals likely to be living in prisons using data on sources of income and health insurance. We used these 2 criteria because current laws restrict adults in prison from receiving Medicaid, Social Security, or Supplemental Security Income.^14^ Additionally, while Medicare covers nearly all adults 65 years or older, individuals in prison are less likely to be enrolled in Medicare.^15^ Although adults in prison are allowed to enroll in Medicare (if they are eligible due to age or disability) or have private insurance, most do not because they are not automatically enrolled, and because such programs would not cover costs of care in prison.^16^

Thus, we define adults in prison as individuals 64 years or younger residing in institutional settings without any form of health insurance and not receiving Social Security or Supplemental Security Income or individuals who are 65 years or older residing in institutional settings without Medicare and not receiving Social Security or Supplemental Security Income. This algorithm is deliberately conservative, and we expect to undercount the number of adults in prison, as individuals who have retained some health insurance would be excluded from our sample. This is particularly the case for individuals aged 55 to 64 years, as we do not allow for any health insurance coverage. We also acknowledge that our identified population may include some, but not all, people in jails (used primarily for individuals awaiting trial or serving sentences of <1 year), as there may be some, but not complete, health insurance for this population.^17^ Importantly, however, we believe our identified population is highly unlikely to include a significant number of people living in nursing homes, since individuals in such settings as those younger than 65 years are most likely to have health insurance and those 65 years or older are likely to be enrolled in Medicare.

We also use data from the Corrections Statistical Analysis tool (CSAT), a publicly available database of statistics about the population in prisons, to validate our identification strategy. These data come from the National Prisoner Statistics and the National Corrections Reporting Program and include information such as prison admissions, releases, and year-end prison populations for 1978 to 2021.

### Measures

#### Outcome Measures

Key outcomes of interest are measures of disability. Specifically, we examined whether a respondent had any cognitive difficulty, such as learning, remembering, concentrating, or making decisions; any difficulty with ambulation, such as walking, climbing stairs, reaching, or lifting; any difficulty with independent living, such as purchasing goods, akin to instrumental activities of daily living; and any difficulty with self-care, such as bathing or dressing, akin to activities of daily living.^18^ All measures are asked as yes or no questions. These measures of disability are used to estimate national disability prevalences reported by the US Census Bureau by demographic and geographic characteristics as well as in policy evaluation.

#### Explanatory Variables

As the ACS survey questionnaire to group quarters is abbreviated, we used almost all available demographic information. Specifically, we examined age, sex (male or female), self-reported race (Black or African American, White, or other [including American Indian or Alaska Native, Chinese, Japanese, Other Asian, Pacific Islander, multiracial, or other]) and ethnicity (Hispanic or non-Hispanic), educational level (high school or General Educational Development diploma or higher), marital status (married with spouse present), and US Census region (Northeast, Midwest, West, or South). eMethods 1 and eTables 1 and 2 in Supplement 1 provide additional data collection information, response rates, and survey language used.

### Statistical Analysis

In this repeated cross-sectional study, we first compared the estimated number of adults 55 years or older identified in prisons using our algorithm in the ACS with the number of estimated adults in prison as reported in the CSAT from 2008 to 2019 to benchmark our estimates. To quantify the comparability of estimates by data source, we calculated the mean percentage difference of our estimate to the reported population 55 years or older in the CSAT. We also conducted validation checks for the identification approach (eMethods 2, eFigure, and eTables 3 and 4 in Supplement 1).

Second, we pooled data from all years to describe the demographic characteristics and disability prevalence for the population of interest. For context, we comparatively describe demographic characteristics and disability of community-dwelling adults 55 years and older.

Last, we use logistic regression in a sample of community-dwelling adults 55 years or older and those in prison to estimate the mean probability of reporting difficulty with cognition, independent living, ambulation, or self-care in the sample after adjusting for age, sex, race, educational level, marital status, year, and US Census region. We present the estimated probability and 95% CIs for disability in the sample overall and examine patterns over time.

We also conducted multiple sensitivity analyses. First, we applied an additional criterion to define the adults 55 years or older in prison. Specifically, we required individuals to report that their current residence was in the same place as the year prior (ie, that they had not moved). This approach was likely to restrict our sample more directly to people in prisons rather than jails. Second, we examined the association of prison status with disability stratified by ages 55 to 64 years, 65 years or older, and 75 years or older, as disability prevalences are likely higher among older adults. Finally, because the population in prisons may require nursing home levels of care, we reran the primary analysis comparing those 55 years or older in prison with a cohort of adults 55 years or older who live in community-dwelling or nursing home settings.

All analyses included survey weights to yield corrected SEs and nationally representative estimates while accounting for complex survey design. To calculate differential effects of prison status, we use the method of recycled estimations. All analyses are conducted in Stata MP, version 18 (StataCorp LLC) and were completed from August 25, 2023, to October 18, 2024. Statistical significance was indicated by 2-sided *P* < .05.

## Results

**Figure 1.**
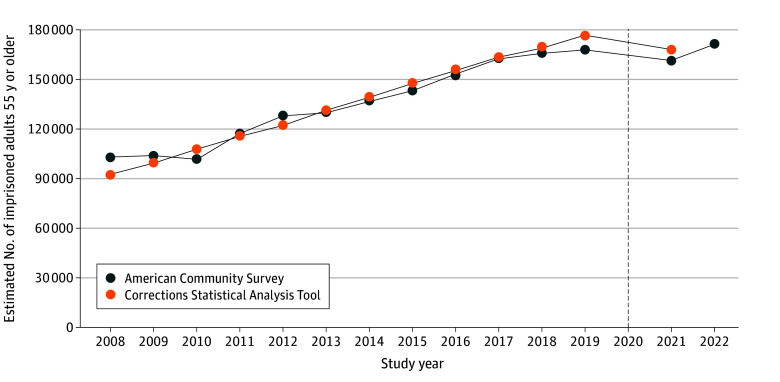
Estimated Number of Adults 55 Years or Older in Prison Data are from the American Community Survey and Bureau of Justice Statistics from 2008 to 2022 (n = 36 623). The Corrections Statistical Analysis Tool only provides data through 2019. The dashed black line indicates the year 2020, when the COVID-19 pandemic began. Estimates from the American Community Survey in year 2020 were excluded due to the experimental nature of the survey weights reflecting the disruption in data collection in 2020.

Figure 1 displays the estimated number of adults 55 years or older in prison using the ACS for years 2008 to 2022 and in the CSAT for years 2008 to 2021. We estimated that the population of adults 55 years or older in prison increased from approximately 102 700 to 171 700 between 2008 and 2022. The estimated numbers of older adults in prison from the ACS were, on average, within 3% of the year-end estimates reported by the CSAT. While the number of older adults in prison grew steadily from 2008 to 2019, it decreased slightly in 2021 compared with 2019, before rebounding slightly in 2022.

A total of 32 623 adults 55 years or older in prison were included in the analysis (mean [SD] age, 62.6 [8.3] years; 3696 [10.36%] female and 28927 [89.64%] male). Of these, 10 441 (32.43%) were Black or African American, 4378 (13.93%) were Hispanic, 18 413 (56.29%) were White, and 3769 (11.28%) were categorized as other. A total of 13 877 665 adults 55 years or older living in the community were included (mean [SD] age, 67.4 [9.5] years). Of these, 1 161 899 (9.83%) were Black or African American, 1 031 258 (9.16%) were Hispanic, 11 501 195 (80.32%) were White, and 1 214 571 (9.85%) were categorized as other.

Compared with their community-dwelling peers, older adults in prison were slightly younger and more likely to be male (89.64% vs 45.98%), not married with spouse present (0 vs 58.33%), Black or African American (32.43% vs 9.83%) or Hispanic (13.93% vs 9.16%); more likely to have less than a high school level of education (71.60% vs 51.71%); and more likely to reside in the South (53.57% vs 37.42%) (Table). These demographic characteristics in the population of adults 55 years or older were consistent over time (see eTable 5 in Supplement 1).

**Table.** Demographic Characteristics of Adults 55 Years or Older by Prison Status^a^

Characteristic	Adults ≥55 y, No. (%)	
	Community dwelling (n = 13 877 665)	In prison (n = 32 623)
Age, mean (SD), y	67.4 (9.5)	62.6 (8.3)
Sex		
Male	6 379 881 (45.98)	28 927 (89.64)
Female	7 497 784 (54.02)	3696 (10.36)
Race		
Black or African American	1 161 899 (9.83)	10 441 (32.43)
White	11 501 195 (80.32)	18 413 (56.29)
Other^b^	1 214 571 (9.85)	3769 (11.28)
Hispanic ethnicity		
No	12 846 407 (90.84)	28 245 (86.07)
Yes	1 031 258 (9.16)	4378 (13.93)
Married with spouse present		
No	5 338 552 (41.67)	32 623 (100)
Yes	8 539 113 (58.33)	0
Highest level of education		
Less than high school	7 165 187 (51.71)	23 332 (71.60)
High school or GED diploma or more	6 712 478 (48.29)	9291 (28.40)
Census region		
Northeast	2 528 157 (18.55)	3330 (11.24)
Midwest	3 084 126 (21.75)	4991 (15.82)
South	5 238 586 (37.42)	17 895 (53.57)
West	3 026 796 (22.28)	6407 (19.37)
Any disability	2 845 288 (21.26)	13 210 (39.39)
Any cognitive difficulty	909 903 (7.13)	7018 (20.84)
Any ambulatory difficulty	2 320 945 (17.33)	9845 (29.30)
Any independent living difficulty	1 393 399 (10.60)	5456 (15.46)
Any self-care difficulty	754 027 (5.83)	3767 (10.45)

Abbreviation: GED, General Educational Development.

^a^ Data are from the American Community Survey, 2008 to 2022.

^b^ Includes American Indian or Alaska Native, Chinese, Japanese, Other Asian, Pacific Islander, multiracial, or other.

**Figure 2.**
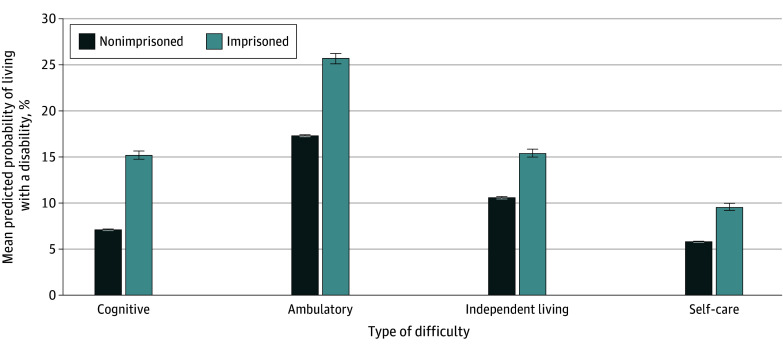
Mean Predicted Probability of Individuals 55 Years or Older With Cognitive Difficulty and Functional Limitations by Prison Status Data are from the American Community Survey from 2009 to 2022, excluding 2020. All model output includes controlling for age, sex, race, highest level of education, marital status, and US Census region. Error bars indicate 95% CIs.

Older adults in prison had higher unadjusted prevalences of disability compared with their community-dwelling peers (Table). On average, we found that adults 55 years or older in prison had higher prevalences of any disability (39.39% vs 21.26%). Most notably, this population was almost 3 times as likely to report cognitive difficulty (20.84% vs 7.13%). These differences persisted after adjusting for demographic characteristics, US Census region, and year fixed effects, with adults 55 years or older in prison still more likely to report cognitive (15.2% [95% CI, 14.8%-15.7%] vs 7.1% [95% CI, 7.1%-7.2%]), ambulatory (25.7% [95% CI, 25.2%-26.3%] vs 17.3% [95% CI, 17.3%-17.4%]), independent living (15.4% [95% CI, 15.0%-15.8%] vs 10.6% [95% CI, 10.6%-10.6%]), and self-care (9.6% [95% CI, 9.2%-10.0%] vs 5.8% [95% CI, 5.8%-5.9%]) difficulties relative to community-dwelling peers (*P* < .05 for all) (Figure 2).

**Figure 3.**
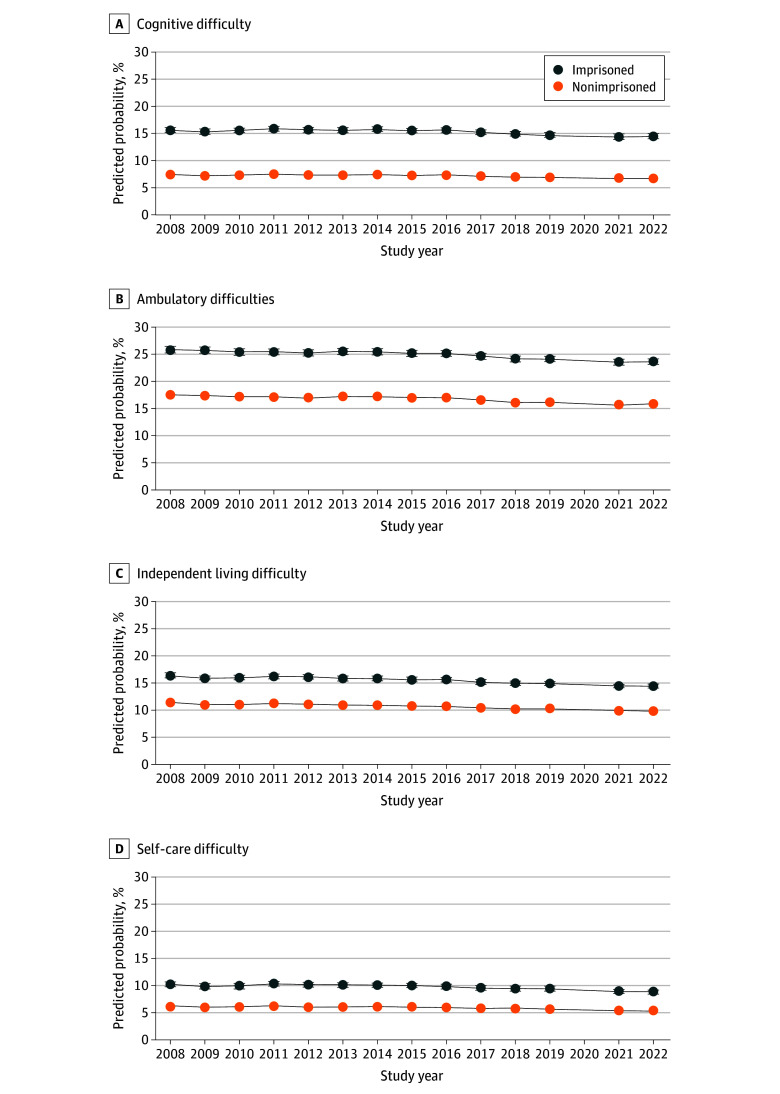
Predicted Probability of Cognitive or Functional Limitations by Prison Status Among Adults 55 Years or Older Data are from the American Community Survey, 2008 to 2022, excluding estimates from 2020. All model output includes controlling for age, sex, race, highest level of education, marital status, and US Census region. Error bars indicate 95% CIs.

When examining patterns over time, we observed the estimated probability of any difficulty with cognition, ambulation, independent living, or self-care was consistently and significantly higher for older adults in prison compared with their community-dwelling peers, after adjusting for demographic characteristics (Figure 3). We observed decreases in probability of disability in the first 2 years of the observation period (2008-2009), but from 2010 through 2022, the probabilities of functional or cognitive disabilities were relatively stable, including during the COVID-19 pandemic (2021-2022).

Results were largely robust to various sample specifications (eTables 6 and 7 in Supplement 1). When restricting the population in prison to those who reported residing in the same residence as the year prior, adults in prison were more likely to report cognitive (14.4% [95% CI, 13.8%-14.9%] vs 7.0% [95% CI, 6.9%-6.9%]), ambulatory (24.5% [95% CI, 23.9%-25.1%] vs 17.0% [95% CI, 17.0%-17.1%]), independent living (14.7% [95% CI, 14.2%-15.2%] vs 10.4% [95% CI, 10.3%-10.4%]), and self-care (8.6% [95% CI, 8.6%-9.5%] vs 5.7% [95% CI, 5.7%-5.7%]) difficulties in adjusted regression models (*P* < .05 for all). Among adults aged 55 to 64 years, those in prisons were more likely to report cognitive and ambulatory difficulties by 3.5 and 2.1 percentage points, respectively, and less likely to report independent living and self-care difficulties by 1.1 and 0.9 percentage points, respectively (*P* < .05 for all). Among adults 65 years or older, those in prisons were more likely to report cognitive difficulties by 14.7 percentage points, to report ambulatory difficulties by 19.9 percentage points, to report independent living difficulties by 16.6 percentage points, and to report self-care difficulties by 14.2 percentage points (*P* < .05 for all). Among adults 75 years or older, those in prisons were more likely to report cognitive difficulties by 24.0 percentage points, ambulatory difficulties by 27.8 percentage points, independent living difficulties by 31.1 percentage points, and self-care difficulties by 29.0 percentage points (*P* < .05 for all). In a sensitivity analysis comparing adults in prison to adults living in community or nursing home settings, we found differences in probability of disability were almost identical to the primary results.

## Discussion

In this cross-sectional study, we describe a novel approach to identifying older adults in prison using nationally representative survey data. We found that the number of older adults likely in prison exhibited steady growth over our sample period, by 67% between 2008 and 2022. However, the growth slowed after the COVID-19 pandemic (2021-2022). We also found that adults 55 years or older in prisons had notably higher prevalences of cognitive and functional limitations than community-dwelling peers, and greater age amplified these differences in disability. While we found disability prevalence to be consistent over time, including after COVID-19 onset, our study underscores the critical need for policies and care models to meet the needs of the growing population of older adults living in prisons with disability. To our knowledge, this study is the largest to date in the US of the prison population 55 years and older and provides insights to the prevalence and change in disability prevalences during the pandemic.

The striking disparity in cognitive limitations between incarcerated and community-dwelling older adults uncovered in our study should motivate a need for programs that focus on this population. Evidence from smaller and nonrepresentative samples of the population in prisons^19^ suggests that cognitive impairment is associated with worse health outcomes, and more emergency department visits, as aligned with evidence from the civilian population. In recent years, some state prisons have developed programs to specifically support incarcerated persons with dementia, such as peer-support programs that pair incarcerated persons with dementia with companions^2,7,20^ or separate units in which staff are prepared to work with cognitively impaired individuals.^21^ For example, the Federal Medical Center Devens in Massachusetts opened a unit based on the nursing home model to serve adults with dementia by an interdisciplinary team and the use of de-escalation techniques.^7^ Given the cost and difficulty in meeting the needs of persons living in prisons and the low rates of recidivism among older populations generally^22^ and populations with functional difficulty more specifically, our findings may also motivate policymakers to consider changes to compassionate release programs for certain populations.

Similarly, as the prison population ages and a growing number of adults die in prison due to illness (eg, heart disease, cancer), there is a need to develop national standards for compassionate models of end-of-life care and models to address functional needs.^23^ In the absence of a national strategy to support the care needs of people aging in prisons, prisons have developed and implemented a patchwork of models to address these needs. For example, in 1988 the Louisiana State Penitentiary in Angola began the nation’s first hospice program in a prison, which has since been adopted by at least 75 other correctional institutions.^2^ Some prisons have invested in creating supportive environments, such as making cells wheelchair accessible.^17^ However, little research examines the effectiveness of such programs on health care use, costs, and patient outcomes.

Our consistent findings of disability prevalence after the COVID-19 pandemic compared with prepandemic years may be explained due to population compositional changes. Older adults are vulnerable to higher morbidity and mortality due to COVID-19. Indeed, observed high rates of death in prisons during the COVID-19 pandemic alongside growth in compassionate release from federal prisons may have shifted the population in prison toward those in better health. Future research is needed to disentangle the effects of the COVID-19 pandemic from temporal trends as well as potential nonresponse bias.

### Limitations

Our study is subject to several limitations. First, individuals with severe impairments in prisons may be less represented in the survey-based research due to lack of a proxy respondent, which would lead to underestimates of the true prevalence of cognitive and functional limitations. Second, lack of trust in research could impede data collection efforts. Third, our proposed algorithm is subject to measurement error and may misclassify individuals, likely underestimating the number of adults 55 years and older in prisons, with our findings thus representing a conservative approach. Fourth, we are limited to the measures of socioeconomic characteristics and disability available in the ACS and therefore are unable to discern severity of impairment by types of disability. Last, the question text is not always well suited for assessing disability in the prison setting: for example, the question about having difficulty with independent living asks about difficulty doing errands alone such as visiting a clinician’s office or shopping. Improved data sources to study this vulnerable population are necessary to overcome this limitation and understand the potential impacts on community health care systems if adults in prison with high care needs transition to receive care in the community.

## Conclusions

Results of this cross-sectional study suggest that adults in prison are more likely to report disability than those in community settings, with the largest differences in cognitive difficulties. These differences persisted during the COVID-19 pandemic. These findings reinforce the importance of better understanding and scaling effective models of geriatric and palliative care within prisons. This study also reveals the need for more regular and detailed data collection to enable monitoring the prevalence and intensity of care needs, as well as for additional research examining effectiveness of models of care and supports designed to meet the care needs of the prison population.

## Notes

Supplement 1. **eMethods 1.** Data Accrual **eTable 1.** Number of Surveys Collected Per Year **eTable 2.** American Community Survey Response Rates (2008-2022) **eMethods 2.** Estimation and Characterization of Nursing Home Population **eFigure.** Estimated Number of Nursing Home Residents 55 Years and Older **eTable 3.** Demographic Statistics of the Likely Nursing Home Resident Cohort 55 Years and Older, 2019 **eTable 4.** States with Highest Prevalences of Imprisonment per 100 000 People, 2019 **eTable 5.** Demographics Over Time of Population 55 Years and Older in Prison **eTable 6.** Predicted Probability Disability **eTable 7.** Differential Effects of Prison Status Across Sensitivity Analyses
eReferences

Supplement 2.
Data Sharing Statement

## References

[1] Pew Charitable Trusts. State prisons and the delivery of hospital care. July 19, 2018. Accessed October 18, 2024. https://www.pewtrusts.org/en/research-and-analysis/reports/2018/07/19/state-prisons-and-the-delivery-of-hospital-care

[2] Hawryluk M. Death and redemption in an American prison. KFF Health News. February 21, 2024. Accessed October 18, 2024. https://www.npr.org/sections/health-shots/2024/02/19/1231119824/prison-hospice-angola-louisiana-quilting

[3] Williams BA, Goodwin JS, Baillargeon J, Ahalt C, Walter LC. Addressing the aging crisis in U.S. criminal justice health care. J Am Geriatr Soc. 2012;60(6):1150-1156. doi:10.1111/j.1532-5415.2012.03962.x 22642489 PMC3374923

[4] McKillop M, Boucher A. Aging prison populations drive up costs. Pew Trusts. February 20, 2018. Accessed October 18, 2024. https://www.pewtrusts.org/en/research-and-analysis/articles/2018/02/20/aging-prison-populations-drive-up-costs

[5] Bor JS. The aging of the US prison population: a public health crisis. Health Aff (Millwood). 2022;41(5):622-627. doi:10.1377/hlthaff.2022.00280 35500173

[6] Ahalt C, Trestman RL, Rich JD, Greifinger RB, Williams BA. Paying the price: the pressing need for quality, cost, and outcomes data to improve correctional health care for older prisoners. J Am Geriatr Soc. 2013;61(11):2013-2019. doi:10.1111/jgs.12510 24219203 PMC3984258

[7] Engelhart K. I’ve reported on dementia for years, and one image of a prisoner keeps haunting me. *New York Times*. August 11, 2023. Accessed October 18, 2024. https://www.nytimes.com/2023/08/11/opinion/dementia-prisons.html

[8] Mitka M. Aging prisoners stressing health care system. JAMA. 2004;292(4):423-424. doi:10.1001/jama.284.4.423 15280325

[9] Valentino-DeVries J. As the pandemic swept America, deaths in prisons rose nearly 50 percent. *New York Times*. February 19, 2023. Accessed October 18, 2024. https://www.nytimes.com/2023/02/19/us/covid-prison-deaths.html

[10] James JE, Foe M, Desai R, Rangan A, Price M. COVID-19 and the reimaging of compassionate release. Int J Prison Health. 2023;19(1):20-34. doi:10.1108/IJPH-08-2021-0072 35730723 PMC10134411

[11] Glaze L. Methodology: survey of prison inmates, 2016. US Department of Justice, Office of Justice Programs, Bureau of Justice Statistics. July 2019. Accessed October 18, 2024. https://bjs.ojp.gov/content/pub/pdf/mspi16.pdf

[12] Skarupski KA, Gross A, Schrack JA, Deal JA, Eber GB. The health of America’s aging prison population. Epidemiol Rev. 2018;40(1):157-165. doi:10.1093/epirev/mxx020 29584869 PMC5982810

[13] U.S. census data for social, economic, and health research. IPUMS. Accessed August 2024. https://usa.ipums.org/usa

[14] Social Security Administration. What prisoners need to know. January 2023. Accessed October 18, 2024. https://www.ssa.gov/pubs/EN-05-10133.pdf

[15] Feinberg R, McKay T, Green J, Bir A; US Department of Health and Human Services. Aging, reentry, and health coverage: barriers to Medicare and Medicaid for older reentrants. February 28, 2018. Accessed October 18, 2024. https://aspe.hhs.gov/reports/aging-reentry-health-coverage-barriers-medicare-medicaid-older-reentrants-0

[16] Centers for Medicare & Medicaid Services. Incarcerated Medicare beneficiaries. Modified September 10, 2024. Accessed October 18, 2024. https://www.cms.gov/training-education/look-up-topics/special-populations/incarcerated-medicare-beneficiaries

[17] Bureau of Justice Statistics. Correctional institutions. Accessed October 18, 2024. https://bjs.ojp.gov/topics/corrections/correctional-institutions

[18] Katz S. Assessing self-maintenance: activities of daily living, mobility, and instrumental activities of daily living. J Am Geriatr Soc. 1983;31(12):721-727. doi:10.1111/j.1532-5415.1983.tb03391.x 6418786

[19] Ahalt C, Stijacic-Cenzer I, Miller BL, Rosen HJ, Barnes DE, Williams BA. Cognition and incarceration: cognitive impairment and its associated outcomes in older adults in jail. J Am Geriatr Soc. 2018;66(11):2065-2071. doi:10.1111/jgs.15521 30232805 PMC6512774

[20] Anderson M. The US prison population is rapidly graying. prisons aren’t built for what’s coming. National Public Radio. March 11, 2024. Accessed October 18, 2024. https://www.northcountrypublicradio.org/news/npr/1234655082/the-u-s-prison-population-is-rapidly-graying-prisons-aren-t-built-for-what-s-coming

[21] Kodama L, Williams B, Morris NP. Prioritizing diversion and decarceration of people with dementia. AMA J Ethics. 2023;25(10):E783-E790. doi:10.1001/amajethics.2023.783 37801064 PMC10962276

[22] Rakes S, Prost SG, Tripodi SJ. Recidivism among older adults: correlates of prison re-entry. Justice Policy Journal. 2018;15(1):1-15.

[23] Carson EA. Mortality in State and Federal Prisons, 2001-2019—Statistical Tables. US Department of Justice, Bureau of Justice Statistics; 2021.

