# Supplementary material for: Prevalence of Disability Among Older Adults in Prison
Source: JAMA Netw Open. 2024 Dec 27;7(12):e2452334. doi: 10.1001/jamanetworkopen.2024.52334 (PMC11681372; doi:10.1001/jamanetworkopen.2024.52334)
Supplement: Supplement 1. — eMethods 1. Data Accrual eTable 1. Number of Surveys Collected Per Year eTable 2. American Community Survey Response Rates (2008-2022) eMethods 2. Estimation and Characterization of Nursing Home Population eFigure. Estimated Number of Nursing Home Residents 55 Years and Older eTable 3. Demographic Statistics of the Likely Nursing Home Resident Cohort 55 Years and Older, 2019 eTable 4. States with Highest Prevalences of Imprisonment per 100 000 People, 2019 eTable 5. Demographics Over Time of Population 55 Years and Older in Prison eTable 6. Predicted Probability Disability eTable 7. Differential Effects of Prison Status Across Sensitivity Analyses eReferences [file jamanetwopen-e2452334-s001.pdf]

Supplementary Online Content

Miller KEM, Shen K, Yang Y, Williams BA, Wolff JL. Prevalence of disability among older adults in prison. *JAMA Netw Open*. 2024;7(12):e2452334.
doi:10.1001/jamanetworkopen.2024.52334

eMethods 1. Data Accrual

eTable 1. Number of Surveys Collected Per Year

eTable 2. American Community Survey Response Rates (2008-2022)

eMethods 2. Estimation and Characterization of Nursing Home Population

eFigure. Estimated Number of Nursing Home Residents 55 Years and Older

eTable 3. Demographic Statistics of the Likely Nursing Home Resident Cohort 55 Years and Older, 2019

eTable 4. States with Highest Prevalences of Imprisonment per 100 000 People, 2019

eTable 5. Demographics Over Time of Population 55 Years and Older in Prison

eTable 6. Predicted Probability Disability

eTable 7. Differential Effects of Prison Status Across Sensitivity Analyses

eReferences

This supplementary material has been provided by the authors to give readers additional information about their work.

© 2024 Miller KEM et al. *JAMA Network Open*.

eMethods 1. Data Accrual

The American Community Survey (ACS) captures numerous sociodemographic, economic, employment and geographic characteristics of adults in the United States through a multi-phase sampling technique. For community-dwelling adults, the ACS is administered through in-person interviews, telephones, mail and the internet. For group-dwelling adults, the ACS is administered through in-person interviews, telephone, or mail (although an online guide is available). For individuals in group quarters, the sampling frame is multifaceted as it includes sampling on the facility level and then within each facility as outlined in the ACS and PRCS Design and Methodology (Version 3.0) – Chapter 8: Data Collection and Capture for Group Quarters. To summarize, facilities are sampled to be representative at the state and federal level through interviews with facility representative(s). Upon agreeing to participation, individuals within facilities are identified and recruited for survey completion. In recent years (2021-2023), the ACS has selected approximately 3.5 million household address and completed final interviews with approximately 2 million households. Among group quarters, approximately 153,000 were selected in the initial sample in 2023 and approximately 129,000 completed final interviews. Please see eTable 1 for sample sizes during the available study period.

© 2024 Miller KEM et al. *JAMA Network Open*.

eTable 1. Number of Surveys Collected Per Year

Year	Initial Addresses Selected	Final Interviews	Initial Sample Selected	Final Actual Interviews	Final Synthetic Interviews*
**2023**	3,544,357	1,978,704	152,993	129,481	144,762
**2022**	3,538,392	1,980,550	150,169	124,846	150,186
**2021**	3,538,442	1,950,832	140,036	119,144	122,876
**2020**	2,872,402	1,406,935	96,955	80,030	147,632
**2019**	3,544,301	2,059,945	167,187	150,305	128,498
**2018**	3,544,000	2,143,000	166,000	150,000	133,000
**2017**	3,526,808	2,145,639	168,590	157,721	133,564
**2016**	3,527,047	2,229,872	206,415	160,572	131,915
**2015**	3,540,307	2,305,707	206,630	161,865	134,224
**2014**	3,540,532	2,322,722	207,403	165,116	129,913
**2013**	3,551,227	2,208,513	207,410	163,663	135,758
**2012**	3,539,552	2,375,715	208,551	154,182	137,086
**2011**	3,272,520	2,128,104	204,553	148,486	150,052
**2010**	2,899,676	1,917,799	197,045	144,948	N/A
**2009**	2,897,256	1,917,748	198,808	146,716	N/A
**2008**	2,894,711	1,931,955	186,862	145,974	N/A

*Please note the synthetic interview numbers include imputation to address sub-state geography estimates and this change in methodology is not expected to impact state-level estimates.

While the ACS has a high response rate, over time response rates have decreased (see eTable 2). Among community-dwelling adults, the response rate is >85% except during 2020 when it was 71%. Among adults in group-quarters, the response rate is >75% except in 2020 when it was 47%. The lower response rates in 2020 are why the survey weights are considered experimental. We exclude 2020 in our analysis due to lack of confidence in the representativeness of the data collected that year.

© 2024 Miller KEM et al. *JAMA Network Open*.

eTable 2. American Community Survey Response Rates (2008-2022)

Year	Response Rate for Households	Response Rate for Group Quarters
2022	84.4	81.2
2021	85.3	74.8
2020	71.2	47.2
2019	86	90.9
2018	92	91.4
2017	93.7	94.7
2016	94.7	95.7
2015	95.8	95.3
2014	96.7	95.9
2013	89.9	95.2
2012	97.3	95.1
2011	97.6	96.9
2010	97.5	97.6
2009	98	98
2008	97.9	98

Please see survey question language below capturing disability for ACS respondents.

[Cognitive Difficulty] *18 a) Because of a physical, mental, or emotional condition, does this person have serious difficulty concentrating, remembering, or making decisions?*

[ ] Yes

[ ] No

[Ambulatory Difficulty] *18 b) Does this person have serious difficulty walking or climbing stairs?*

[ ] Yes

[ ] No

[Self-Care Difficulties] *18 c. Do you have difficulty dressing or bathing?*

© 2024 Miller KEM et al. *JAMA Network Open*.

[ ] Yes

[ ] No

Answer question 19 if you are 15 years old or over. Otherwise, SKIP to on page 7 for further instructions; do not answer any more questions.

[Independent Living Difficulties] *19. Because of a physical, mental, or emotional condition, do you have difficulty doing errands alone such as visiting a doctor's office or shopping?*

[ ] Yes

[ ] No

© 2024 Miller KEM et al. *JAMA Network Open*.

eMethods 2. Estimation and Characterization of Nursing Home Population

While relatively little is known about the socio-demographic characteristics and health status of the population in prison, the nursing home population is well characterized. We thus comparatively examine the estimated number of nursing home residents and their demographic characteristics to other estimates of the nursing home population to validate our approach.

We first estimate the number of likely nursing home residents and compare our American Community Survey (ACS) estimates to the national estimates provided by the American Community Survey, see the eFigure. For context, in 2019, the number of nursing facility/skilled nursing facility respondents aged 55 and older was reported to be 1,396,539 in ACS tables released by the Census Bureau(4). Using our approach, we estimate approximately 1,508,429. Thus, our classification approach overcounts likely nursing home residents and thus *undercounts* individuals in prison.

Second, we examine demographic and functional characteristics of the likely nursing home residents to determine if the sample characteristics are aligned with existing evidence about the nursing home population. See eTable 3. As aligned with existing evidence, in 2019, we observe the likely nursing home resident population is approximately 60% female, 78% white, and the majority of residents have difficulty with independent living, self-care or ambulation, as aligned with expectations and existing literature (5).

Finally, we also examine the prevalences of adults in prison per capita at the state level to determine if the states with the highest prevalences of adults aged 55+ in prison per capita are like states with the highest prevalences of imprisonment (of any age) per capita. We observe 7 of the 10 states with the highest prevalences of imprisonment per capita in 2019, as reported by the Bureau of Justice Statistics, also have some of the highest prevalences of imprisonment among adults 55+ per capita (see eTable 4).

© 2024 Miller KEM et al. *JAMA Network Open*.

Based on these descriptive statistics, we conclude that our algorithm is distinguishing between individuals aged 55+ who reside in a nursing home versus those residing in prisons, but likely undercounts the population in prison.

© 2024 Miller KEM et al. *JAMA Network Open*.

eFigure. Estimated Number of Nursing Home Residents 55 Years and Older

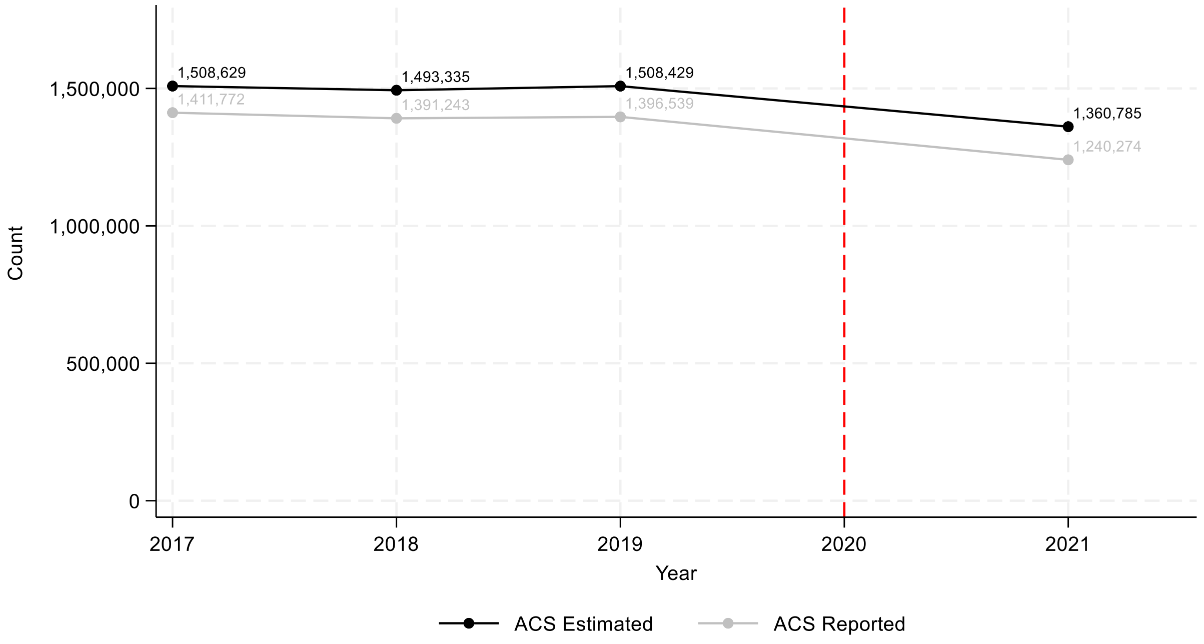

SOURCES: American Community Survey (ACS).

© 2024 Miller KEM et al. *JAMA Network Open*.

eTable 3. Demographic Statistics of the Likely Nursing Home Resident Cohort 55 Years and Older, 2019

	**Likely Nursing Home Residents aged 55+**
	N=1,508,429[1]
Age, mean (SD)	77.85 (11.85)
Sex	
Male	41.01%
Female	58.99%
Race	
White	77.93%
Black/African American	16.51%
American Indian or Alaska Native, Chinese, Japanese, Other Asian or Pacific Islander, Two or more race or Other race	5.56%
Highest Level of Education	
Less than high school	70.06%
High School/GED or more	29.94%
Census Region	
Northeast	22.65%
Midwest	28.18%
South	34.29%
West	14.87%
Any cognitive difficulty	56.82%
Any ambulatory difficulty	80.13%
Any Independent living difficulty	82.55%

© 2024 Miller KEM et al. *JAMA Network Open*.

Any Self-care Difficulty	72.30%

[1]n (weighted) (%)

© 2024 Miller KEM et al. *JAMA Network Open*.

eTable 4. States with Highest Prevalences of Imprisonment per 100 000 People, 2019

State	Prevalence of imprisonment for age 55+	Order of Prevalence of imprisonment for age 55+	Prevalence of imprisonment for ages 18+	Order of Prevalence of imprisonment for ages 18+
Texas	547.7	1	709	6
Oklahoma	538.9	2	840	2
Arkansas	509.0	3	762	4
Georgia	489.1	4	663	8
Alaska	485.2	5	323	37
Mississippi	451.9	6	830	3
Florida	425.3	7	552	14
Alabama	415.7	8	539	17
Louisiana	403.3	9	887	1
Arizona	347.3	10	719	5

Source: Prevalence of Imprisonment for ages 55 and older are author tabulation based on American Community Survey. Prevalence of imprisonment for all ages are from the Bureau of Justice Statistics (https://bjs.ojp.gov/content/pub/pdf/p19.pdf).

© 2024 Miller KEM et al. *JAMA Network Open*.

eTable 5. Demographics Over Time of Population 55 Years and Older in Prison

	**2010-2011**<br>N=3,468[1]	**2018-2019**<br>N=5,587[1]	**2021-2022**<br>N=7,166[1]
Age, mean (SD)	62.1 (8.2)	62.4 (7.9)	63.2 (8.1)
Sex	3,091 (90.09%)	5,041 (91.73%)	6,389 (90.52%)
Male	377 (9.91%)	546 (8.27%)	777 (9.48%)
Female			
	2,048 (58.24%)	3,177 (56.71%)	3,558 (49.43%)
Race	1,122 (32.59%)	1,878 (33.87%)	2,267 (31.77%)
White	298 (9.18%)	532 (9.43%)	1,341 (18.80%)
Black/African American			
American Indian or Alaska Native, Chinese, Japanese, Other Asian or Pacific Islander, Two or more race or Other race	2,414 (69.31%)	3,958 (71.54%)	5,359 (75.09%)
	1,054 (30.69%)	1,629 (28.46%)	1,807 (24.91%)
Highest Level of Education			
Less than high school	476 (14.61%)	551 (10.63%)	560 (9.53%)
High School/GED or more	579 (17.76%)	825 (14.62%)	936 (14.42%)
	1,687 (48.79%)	3,178 (55.83%)	4,359 (58.03%)
Census Region	726 (18.85%)	1,033 (18.93%)	1,311 (18.01%)
Northeast	62.1 (8.2)	62.4 (7.9)	63.2 (8.1)
Midwest			
South	3,091 (90.09%)	5,041 (91.73%)	6,389 (90.52%)
West	377 (9.91%)	546 (8.27%)	777 (9.48%)

[1]Mean (SD); n (unweighted) (%)

© 2024 Miller KEM et al. *JAMA Network Open*.

eTable 6. Predicted Probability Disability

	Predicted Probability (Standard Error)			
	Any Cognitive Difficulty	Ambulatory Difficulty	Independent Living Difficulty	Self-Care Difficulty
**More Expansive Comparison Group**				
Community-Dwelling and Nursing Home Residents	0.083***	0.186***	0.121***	0.072***
	(0.000)	(0.000)	(0.000)	(0.000)
In prison	0.150***	0.256***	0.149***	0.093***
	(0.002)	(0.003)	(0.002)	(0.002)

* $p < 0.05$, ** $p < 0.01$, *** $p < 0.001$

All model output includes controlling for age, sex, race, highest level of education, marital status, and Census region.

© 2024 Miller KEM et al. *JAMA Network Open*.

eTable 7. Differential Effects of Prison Status Across Sensitivity Analyses

	Any Cognitive Difficulty	Ambulatory Difficulty	Independent Living Difficulty	Self-Care Difficulty
**Under Age 65**				
Community-Dwelling	-ref-	-ref-	-ref-	-ref-
In prison	0.035***	0.021***	-0.011***	-0.009***
	(0.002)	(0.002)	(0.001)	(0.001)
N	6142241	6142241	6142241	6142241
**Age 65+ Only**				
Community-Dwelling	-ref-	-ref-	-ref-	-ref-
In prison	0.147***	0.199***	0.166***	0.142***
	(0.004)	(0.007)	(0.005)	(0.005)
N	7768047	7768047	7768047	7768047
**Age 75+ Only**				
Community-Dwelling	-ref-	-ref-	-ref-	-ref-
In prison	0.240***	0.278***	0.311***	0.290***
	(0.009)	(0.013)	(0.012)	(0.012)
N	3242052	3242052	3242052	3242052
**More Restricted Definition of Adult in Prison (live in same place as last year)**				
Community-Dwelling	-ref-	-ref-	-ref-	-ref-
In prison	0.035***	0.021***	-0.011***	-0.009 ***
	(0.002)	(0.002)	(0.001)	(0.001)
N	6,142,241	6,142,241	6,142,241	6,142,241

* $p < 0.05$, ** $p < 0.01$, *** $p < 0.001$

All model output includes controlling for age, sex, race, highest level of education, marital status, and Census region.

© 2024 Miller KEM et al. *JAMA Network Open*.

eReferences

1. U.S. Census Bureau. *American Community Survey: Design and Methodology Report*. 2022. Available at: https://www2.census.gov/programs-surveys/acs/methodology/design_and_methodology/2022/acs_design_methodology_report_2022.pdf.

2. U.S. Census Bureau. *American Community Survey (ACS) Response Rates*. Available at: https://www.census.gov/acs/www/methodology/sample-size-and-data-quality/response-rates/. Accessed October 27, 2024.

3. U.S. Census Bureau. *American Community Survey (ACS) Sample Size*. Available at: https://www.census.gov/acs/www/methodology/sample-size-and-data-quality/sample-size/index.php. Accessed October 28, 2024.

4. US Census Bureau. Group Quarters Type (3 Types) by Sex by Age. US Census Bureau; 2019.

5. National Academies of Sciences, Engineering, and Medicine. Medicine. The National Imperative to Improve Nursing Home Quality: Honoring Our Commitment to Residents, Families, and Staff. The National Academies Press; 2022.

© 2024 Miller KEM et al. *JAMA Network Open*.
