# Supplementary material for: Prevalence of Disability Among Older Adults in Prison
Source: JAMA Netw Open. 2024 Dec 27;7(12):e2452334. doi: 10.1001/jamanetworkopen.2024.52334 (PMC11681372; doi:10.1001/jamanetworkopen.2024.52334)
Supplement: Supplement 2. — Data Sharing Statement [file jamanetwopen-e2452334-s002.pdf]

Data Sharing Statement

Miller. Prevalence of Disability Among Older Adults in Prison. *JAMA Netw Open*. Published December 27, 2024. doi:10.1001/jamanetworkopen.2024.52334

Data
Data available: No

Additional Information
Explanation for why data not available: Due to the terms of use, I am not able to share the source dataset. However, the data is publicly available and through GitHub I will provide a link to the data I used and will make available all code used to construct the analytic file and run all analyses.
